# Understanding Social Cognition Using Virtual Reality: Are We still Nibbling around the Edges?

**DOI:** 10.3390/brainsci10010017

**Published:** 2019-12-28

**Authors:** Rocco Salvatore Calabrò, Antonino Naro

**Affiliations:** IRCCS Centro Neurolesi Bonino Pulejo, 98124 Messina, Italy; antonino.naro@irccsme.it

**Keywords:** virtual reality, social cognition, social neuroscience, sense of presence, motor-cognitive rehabilitation

## Abstract

Virtual Reality (VR) has a variety of applications in various fields of study, including social work and human performance training. Useful information regarding the neurobiological underpinnings of social cognition (SC) has been obtained from the use of VR. This was mainly achieved by substituting the use of simple and static stimuli (that lack many of the potentially important aspects of real-world activities and social interactions) with fully interactive, three-dimensional computerized models of social situations that can be fully controlled by the experimenter, and can simulate a real-world setting as recently pointed out by Parsons et al. (Virtual Reality for Research in Social Neuroscience. Brain Sciences, 2017). As a consequence, the cognitive training in the field of SC and, broadly, social neuroscience, has greatly benefited from the use of VR. However, specific issues concerning the VR neurophysiological underpinnings remain to be clarified, as well as the social and cultural consequences of VR technologies focusing on the processing of social information and the consequences arising from the understanding of self and others. Notwithstanding, it is important to remark that VR-based social neuroscience scenarios can reliably enhance the affective experience and social interactions, whether added to or coupled with traditional cognitive behavioural therapy.

Since the late 1980–90s, virtual reality (VR) has developed and is increasingly used in a great bulk of human activities and scientific fields, including business, commerce, education, entertainment, and health. Furthermore, VR represents one of the most promising technologies for neurorehabilitation, beyond robots, brain-computer interfaces, wearable devices for human movement analysis, noninvasive brain stimulators, neuroprostheses, and computers/tablets for electronic clinical records and planning [1]. The usefulness and feasibility of VR to such heterogeneous fields arise from its definition, i.e., an “advanced form of human-computer interface that allows the user to interact with and become immersed in a computer-generated environment in a naturalistic fashion” [2]. In other words, computers elaborate a simulated world from the real world using real-time graphics, through which the subject can view and interact with the environment (thanks to interface devices and body tracking sensors) [3]. Consequently, the user can be provided with a fully immersive and a deep sense of presence within the environment [2,3], i.e., when the “the individual perceives himself or herself to be enveloped by, included in, and interacting with an environment that provides a continuous stream of stimuli” [4]. In other words, the brain automatically interprets the immersion as being real even though it is consciously understood to be virtual (namely, a sense of ecological validity) [5].

This last issue is of utmost importance in the study of social cognition (SC), which represents a specific focus of social neuroscience [5]. SC can be defined as “the study of mental processes involved in perceiving, attending to, remembering, thinking about, and making sense of the people in our social world”. This study was carried out using a set of socio-cognitive processes that allows subjects to create a representation of the social world around them [6,7]. The use of VR in social neuroscience circumvented the methodological constraints of providing participants with static stimuli to estimate social cognition processes, as recently pointed out by Parsons and coll. [5], concerning, among others, simple phobias, post-traumatic stress disorder, social anxiety, and addiction. 

The meaningfulness of such issues is four-fold. First, it allows studying the neurobiological processes subtending SC in a controlled manner (by using recordings of the intrinsic electrical activity of the brain, brain stimulation, neuroimaging, psychophysiological measurements, biomarkers, and computational modelling), avoiding any external biasing factors. In fact, extrinsic stimuli may not be representative of real-life social encounters, potentially resulting in ceiling effects [5,8]. 

Second, it allows training the patient in a controllable and safe environment, which can augment the representativeness and generalizability of the virtual, to real-world social cognition [5]. Indeed, SC may be compromised in patients with traumatic brain injury, autism, and other neuropsychiatric disorders, and thus rehabilitation of SC by using VR may lead to better functional outcomes and more appropriate management of such patients [9,10]. This is proposed to occur by increasing the sense of presence/social presence experienced by the participant (as they experience emotionally engaging background narratives to enhance the affective experience and social interactions) [11,12]. Moreover, VR allows increased feedback to the central nervous system through intensive, repetitive and task-oriented exercises that are performed in a virtual environment, developing a knowledge of the results and the quality of the movements (i.e., knowledge of the performance), leading to “reinforcement learning” [13]. 

Third, VR permits precisely tracking the participant’s behaviour and coordinate the distinct virtual environment components to obtain a holistic, integrated measurement of target social interactions [5]. 

Last, VR is the unique tool allowing studying social situations that would be otherwise impossible to investigate using conventional research paradigms, including the embodying another self and the role of the mirror neuron system. This last concept plays a significant role in social cognition [14,15]. In fact, mirror neuron system empowers the subject to intuitively feel that others have intentions, sensations and emotions by associating our own motor programs and the sensory consequences of the observation of executing them by others in a Hebbian-like fashion. Also, a mirror neuron system-like mechanism might also support sensation, emotion experimentation and manifestation while witnessing those of others [14,15].

Despite all these positive aspects, the neurophysiological mechanisms through which VR works are known only still partially. Indeed, a better knowledge of the neurophysiological underpinnings of VR may allow tailoring the cognitive-motor training to patient’s needs and goals. The main hypothesis is that VR may entrain the same neural pathways that are involved in motor learning and cognitive processes, thus reinforcing the neuroplasticity processes triggered by motor-cognitive rehabilitation that are fundamental for neural damage recovery [13]. Indeed, VR may magnify the effects of the assisted, intensive, and repetitive motor-cognitive tasks delivered within a rehabilitative setting by providing patients with task-oriented exercises (which are motivating and entraining) and with additional knowledge of performance and results [16,17]. 

Furthermore, the use of VR may cause some problems, especially in those individuals predisposed to develop cybersickness. Cybersickness is a subtype of motion sickness that arises from the immersion in a virtual environment (VE) [18]. Cybersickness and motion sickness show very similar symptoms, including nausea, vomiting, pallor, eructation, salivation, drowsiness, disorientation, dizziness, headaches, the sensation of being in movement, and postural instability [19]. The presence of such symptoms could impair the quality of the virtual experience and reduce compliance and motivation in the virtual rehabilitation field [19]. The pathophysiological mechanisms subtending cybersickness are not completely understood, although the “multisensory mismatch” theory (highlighting the role of an external input inducing conflicts between two or more receptive sensory structures) should be taken into account [19].

To summarize, useful information regarding the neural correlates of social cognition (SC) has been obtained from the use of VR, thus further promoting cognitive training in the field of SC and, broadly, social neuroscience. However, specific issues concerning the VR neurophysiological underpinnings remain to be clarified, as well as the social and cultural consequences of VR technologies focusing on the processing of social information, and the consequences arising from the understanding of self and others. Furthermore, the level of realism provided by VR systems has to be finely tuned as the psychological effects of exposing participants to extreme social situations (e.g., ethical dilemmas) may cause unwanted, and even severe psychological effects [20]. Notwithstanding, even though social neuroscience has extended to investigating real-world social questions via dynamic VR paradigms, it is important to remark that VR-based social neuroscience scenarios can reliably enhance the affective experience and social interactions whether added to or coupled with traditional cognitive behavioural therapy.
